# Vulnerability to Pollen‐Related Asthma Hospital Admissions in the UK Biobank: A Case‐Crossover Study

**DOI:** 10.1111/all.16612

**Published:** 2025-06-03

**Authors:** Elaine Fuertes, Garyfallos Konstantinoudis, Diana van der Plaat, Adam Koczoski, Mikhail Sofiev, Paul Agnew, Lucy Neal, Debbie Jarvis

**Affiliations:** ^1^ National Heart and Lung Institute Imperial College London London UK; ^2^ Grantham Institute for Climate Change and the Environment Imperial College London London UK; ^3^ Finnish Meteorological Institute Helsinki Finland; ^4^ Met Office Exeter UK

**Keywords:** air pollution, asthma, case‐crossover, cohort, pollen

To the Editor,

In 2023, a systematic review and meta‐analysis concluded that outdoor pollen may affect asthma exacerbations in adults [1]. However, the authors cautioned that the literature is heterogenous and most studies are ecological and have not considered individual‐level confounders or effect modifiers.

We here examined associations between daily concentrations of grass pollen, three tree pollens, and nettle pollen (represents weeds) assigned to home addresses using a deterministic model developed by the UK Met Office [2], and asthma hospital admissions among UK Biobank adult participants [3] from 2011 to 2022. Effect modification by demographic and environmental factors was examined.

Bidirectional adjusted time‐stratified case‐crossover models assessed associations between emergency asthma hospital admissions (493 ICD9 or J45/J46 ICD10 codes) and same‐day pollen levels (high vs. low) [2] assigned to home addresses. Further information on data sources and the statistical analysis is in the Supporting Information.

Effect modification by daily mean NO_2_, PM_2.5_, and ozone was examined (using tertiles), and models were stratified by annual average NO_2_ and PM_2.5_ levels, % greenspace, sex, age, ethnicity, BMI, smoking, education, income, deprivation (as defined in Table S1), and genetic risk for atopy, the latter as a marker for sensitization (Supporting Information).

There were 1893 asthma emergency hospital admissions (primary diagnosis, > 14 days apart) among 1489 participants from January to September 2011–2022. Descriptive statistics for the pollen variables, study population, and confounders (i.e., meteorological and pollution variables) are provided in Table 1; Tables S1 and S2, respectively.

**TABLE 1:** Days with asthma events and high pollen levels (January to September, 2011–2022), and associations between asthma hospital admissions and same day pollen levels (high vs. low).

Data source	Pollen	Days with asthma events	% days with high pollen levels^a^	OR [95% CI]^b^
UK Met office	Alder	1893	3.8	1.51 [1.10, 2.09]
	Birch	1893	4.0	0.93 [0.68, 1.27]
	Grass	1893	12.1	1.31 [1.00, 1.70]
	Oak	1893	2.6	1.08 [0.75, 1.56]
	Nettle	1893	10.2	0.99 [0.78, 1.25]
	Overall	1893	26.6	1.17 [0.99, 1.37]
European pollen reanalysis	Alder^c^	1195	5.6	1.35 [0.97, 1.88]
	Birch^d^	838	16.8	0.90 [0.69, 1.18]

^a^ The cutoff for high days was ≥ 30 grains/m^3^ for all pollens except ≥ 40 grains/m^3^ was used for birch and nettle. The “overall” pollen variable was defined as high if any of the individual pollens were high on a given day.

^b^ Odds ratios [95% confidence intervals] derived from conditional logistic models adjusted for daily mean temperature, precipitation, relative humidity, windspeed, and weekends/public holidays.

^c^ Only data from January to May are available, used for external validation.

^d^ Only data from March to June are available, used for external validation.

Asthma hospital admissions were higher on days of high alder and grass concentrations and when considering all pollen types together (Table 1). These findings remained fairly consistent when adjusting for air pollutants (Table S3), examining different lags (Table S4) and restricting to groups and time periods of interest (Table S5). Effect estimates for alder (but not birch) were also elevated when using a second pollen dataset for external validation (European pollen reanalysis, not available for the other pollen types, Table 1) [4]. The adverse association observed with high grass concentrations is in line with most existing literature, whereas that with alder has been reported in some but not all studies [1].

Associations for several pollens were notably stronger on days with high daily NO_2_ and PM_2.5_ levels (Figure 1), suggesting an acute interactive effect. This observation adds significantly to the currently weak evidence from epidemiological studies of pollution‐pollen interactions, despite robust results from in vitro and experimental work [5].

**FIGURE 1:**
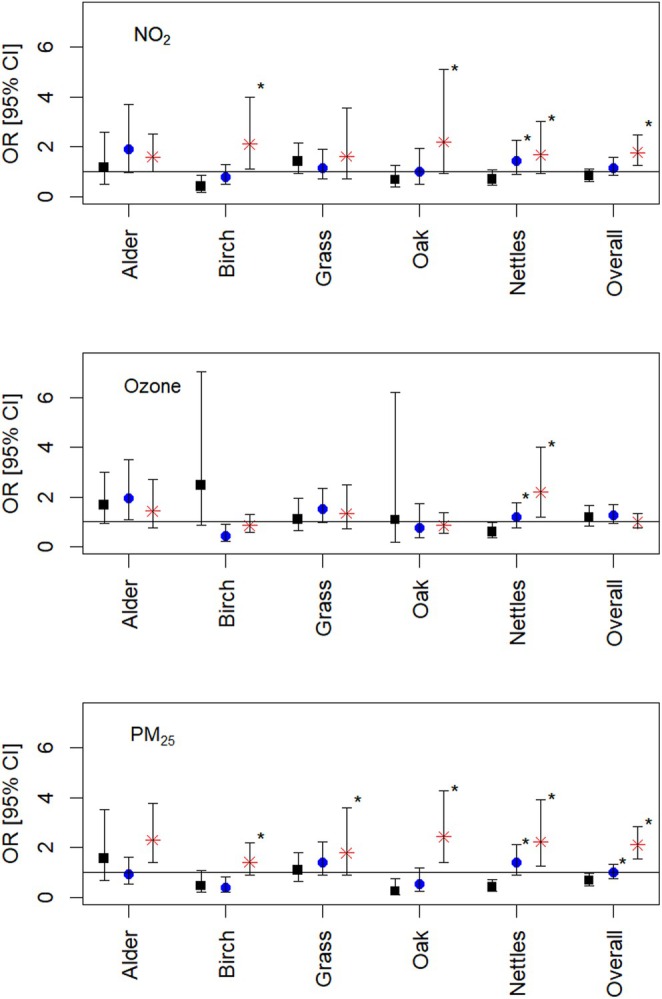
Associations between asthma hospital admissions and pollen levels (high versus low), stratified by tertiles of daily NO_2_, ozone and PM_2.5_ levels (lowest tertile = black squares, middle tertile = blue circles, highest tertile = red stars). *p‐interaction term < 0.05.

For some pollens, higher but imprecise effect estimates were observed among those with a higher genetic risk of atopy, which supports biological plausibility (Figure S1), and among those of Non‐White ethnicity (Figure S2), although sample sizes are small in these groups.

This work capitalizes on large individual‐level health and demographic data which allowed important effect modifiers to be considered. However, we lacked objective measures of sensitization to pollen, residual confounding remains a concern (e.g., no information on medication, pollen avoidance behavior, fungal and other spores, and circulating viruses) and our findings may not be generalizable nationally as participants are clustered around recruitment sites.

We used a highly spatially and temporally resolved pollen dataset to assign six pollen exposures to participant home addresses [2]. This approach resolves the significant problem of the unknown representativeness of point observations. Previous evaluations of the pollen model predictions against the UK observations network show a good level of model skills [2]. Further developments to the pollen model are ongoing, including the production of a UK pollen reanalysis from present day back to 2000.

In conclusion, this analysis suggests exposure to grass and tree (alder) pollen can have serious health impacts for asthma patients, with effects being greatest on high air pollution days. This has important public health implications given that many asthma patients are sensitized and several pollen exposures are predicted to worsen with climate change [6].

## Author Contributions

E.F. and D.J.: conceptualization; E.F.: funding acquisition, visualization, writing – original draft; E.F., G.K., and D.P.: formal analysis; methodology; All authors: data curation, investigation, resources, writing – review and editing.

## Conflicts of Interest

The authors declare no conflicts of interest.

## Supporting information


**Table S1.** Descriptive statistics closest to the first reported asthma hospital admission (*N* = 1489 participants).
**Table S2.** Summary statistics of the daily modeled covariates (January to September, 2011–2022).
**Table S3.** Associations (OR 95% CI) between daily pollen levels and asthma hospital admissions, further adjusted for daily NO_2_, PM_2.5_ mass, and ozone concentrations.
**Table S4.** Associations (OR 95% CI) between daily pollen levels on lag day 1, 2, and 3, as well as cumulative lag from 0 to 3 days, and asthma hospital admissions.
**Table S5.** Associations (OR 95% CI) between daily pollen levels and asthma hospital admissions, restricted to groups and periods of interest.
**Figure S1.** Associations between the pollen types and asthma hospital admissions, stratified by ethnicity (White = black squares, Non‐White = blue circles).
**Figure S2.** Associations between the pollen types and asthma hospital admissions, stratified by the 20th vs. 80th percentiles of a genetic risk score for atopy (less genetic risk = black squares, greater genetic risk = blue circle).

## Data Availability

The data that support the findings of this study are available from the original data owners (under data transfer agreements). Restrictions apply to the availability of these data, which were used under license for this study. Data are available from the author(s) with the permission of the original data owners (under data transfer agreements).
