# Supplementary material for: Vulnerability to Pollen‐Related Asthma Hospital Admissions in the UK Biobank: A Case‐Crossover Study
Source: Allergy. 2025 Jun 3;80(7):2081–3. doi: 10.1111/all.16612 (PMC12261866; doi:10.1111/all.16612)
Supplement: Supplementary file 1 — Table S1. Descriptive statistics closest to the first reported asthma hospital admission (N = 1489 participants). Table S2. Summary statistics of the daily modeled covariates (January to September, 2011–2022). Table S3. Associations (OR 95% CI) between daily pollen levels and asthma hospital admissions, further adjusted for daily NO2, PM2.5 mass, and ozone concentrations. Table S4. Associations (OR 95% CI) between daily pollen levels on lag day 1, 2, and 3, as well as cumulative lag from 0 to 3 days, and asthma hospital admissions. Table S5. Associations (OR 95% CI) between daily pollen levels and asthma hospital admissions, restricted to groups and periods of interest. Figure S1. Associations between the pollen types and asthma hospital admissions, stratified by ethnicity (White = black squares, Non‐White = blue circles). Figure S2. Associations between the pollen types and asthma hospital admissions, stratified by the 20th vs. 80th percentiles of a genetic risk score for atopy (less genetic risk = black squares, greater genetic risk = blue circle). [file ALL-80-2081-s001.docx]

**SUPPORTING INFORMATION**

**Vulnerability to pollen-related asthma hospital admissions in the UK Biobank:**

**a case-crossover study**

Elaine Fuertes^1^, Garyfallos Konstantinoudis^2^, Diana van der Plaat^1^, Adam Koczoski^1^, Mikhail Sofiev^3^, Paul Agnew^4^, Lucy Neal^4^, Debbie Jarvis^1^

1. National Heart and Lung Institute, Imperial College London, UK
2. Grantham Institute for Climate Change and the Environment, Imperial College London, London, UK
3. Finnish Meteorological Institute, Helsinki, Finland
4. Met Office, Exeter, UK

**Pollen:** Hourly mean grain concentrations of alder, birch, grass, oak, and nettle (a marker of weeds) were obtained from the UK Met Office pollen modelling system (0.05^o^ x 0.05^o^ resolution) from 2011-2022. This model was developed as part of the government funded Strategic Priorities Fund Clean Air Project, and details of its development, verification and applications are published^1^. The hourly data were averaged into daily means and dichotomized into low vs high based on > 30 grains/m^3^ for all pollens except birch and nettle for which > 40 grains/m^3^ was used^1^. An ‘overall’ pollen variable was defined as high if any of the individual pollens were high on a given day.

**Analysis:** Associations were modelled using bidirectional time-stratified case-crossover models^2–5^. This approach controls for time-invariant confounding variables (e.g. age, sex, ethnicity) and factors that vary slowly (e.g. deprivation, income) and allows individual-level effect modification to be considered. ‘Case’ days were the day of hospital admission. Control days were the same day of the week, month and year. Models were adjusted for daily mean temperature (mean of daily minimum and maximum), precipitation, relative humidity, wind speed and weekends/public holidays. All variables were mapped to the home address closest in time to the day of hospital admission. A natural spline with 2 degrees of freedom was placed on mean temperature, precipitation and windspeed.

**Derivation of the genetic risk score:** We identified six genome-wide association studies which used an objective measure of sensitization as the outcome^6–11^. These studies yielded 30 independent SNPs (linkage disequilibrium r^2^ < 0.2) that are available in the European Community Respiratory Health Survey II (ECRHS, independent test population^12^). These SNPs were individually regressed against grass sensitization status (IgE levels to grass > 0.35kU/L) in logistic models adjusted for sex, age, centre and four principal components. The risk allele for each SNP was the allele that increased the odds of sensitization in the ECRHS. Individual genetic risk scores were constructed for all UK Biobank participants as the sum of the product of the number of risk alleles (0, 1, or 2) for each SNP and their beta regression estimates from the model in ECRHS which assessed marginal genetic effects.

**Table S1:** Descriptive statistics closest to the first reported asthma hospital admission (N=1,489 participants)

| **Characteristic** |  | **N or mean** | **% or SD** | **UK Biobank data field** |
| --- | --- | --- | --- | --- |
| Sex | Male | 479 | 32.2 | 31 |
| Age at admission  (years, mean, SD) | < 65 | 56.7 | 5.7 | 21003 |
|  | > 65 | 72.1 | 4.0 |  |
| Ethnicity | White | 1333 | 90.7 | 21000 |
|  | Non-White | 137 | 9.3 |  |
| BMI | Normal | 309 | 21.0 | 21001 |
|  | Overweight | 539 | 36.7 |  |
|  | Obese | 621 | 42.3 |  |
| Doctor diagnosed hayfever, allergic rhinitis or eczema |  | 829 | 44.2 | 6152 |
| Smoking status | Never-smoker | 817 | 55.7 | 20116 |
|  | Former smoker | 498 | 33.9 |  |
|  | Current smoker | 152 | 10.4 |  |
| Education level^†^ | Low | 357 | 24.9 | 6138 |
|  | Medium | 352 | 24.6 |  |
|  | High | 724 | 50.5 |  |
| Household income (GBP) | < 31,000 | 779 | 64.8 | 738 |
|  | > 31,000 | 424 | 35.2 |  |
| Index of multiple deprivation^‡^ | Most deprived | 344 | 23.6 | 26410 (England) |
|  | Medium deprived | 438 | 30.1 | 26426 (Wales) |
|  | Least deprived | 674 | 46.3 | 26427 (Scotland) |
| Greenspace 300m around home (%, mean, SD) | Lowest tertile | 12.6 | 4.6 | 24503 |
|  | Middle tertile | 28.1 | 5.3 |  |
|  | Highest tertile | 58.3 | 16.3 |  |
| Annual average NO_2_ (µg/m^3^, mean, SD) | Lowest tertile | 31.8 | 5.4 | 24004 |
|  | Middle tertile | 44.1 | 2.9 |  |
|  | Highest tertile | 62.4 | 15.3 |  |
| Annual average PM_2.5_ mass (µg/m^3^, mean, SD) | Lowest tertile | 9.0 | 0.5 | 24006 |
|  | Middle tertile | 10.0 | 0.2 |  |
|  | Highest tertile | 11.4 | 0.9 |  |

^†^low: none; medium: A-level, O-level of CSEs; high: college, university, NVQ or other professional qualifications

^‡^deprivation coded as within-country tertiles based on all UK Biobank participants

**Table S2:** Summary statistics of the daily modelled covariates (January to September, 2011-2022)

| **Daily variable** | **Min** | **P25** | **Median** | **P75** | **Max** | **Source** |
| --- | --- | --- | --- | --- | --- | --- |
| Mean temperature (^o^C) | -3.8 | 6.3 | 10.7 | 15.2 | 25.9 | UK Met Office^13^ |
| Precipitation (mm) | 0 | 0 | 0.2 | 2.4 | 40.2 | UK Met Office^13^ |
| Relative humidity (%) | 43.5 | 73.4 | 79.7 | 85.7 | 98.9 | ERA5-Land hourly data^14^ |
| Wind speed (m/s) | 0.1 | 2.3 | 3.3 | 4.5 | 10.5 | ERA5-Land hourly data^14^ |
| NO_2_ (µg/m^3^) | 2.0 | 11.3 | 16.9 | 24.1 | 75.7 | UK Met Office, up to 2019^15^ |
| PM_2.5_ mass (µg/m^3^) | 0.9 | 5.8 | 8.5 | 13.9 | 73.5 | UK Met Office, up to 2019^15^ |
| Ozone (µg/m^3^) | 2.7 | 38.9 | 49.0 | 60.0 | 94.4 | UK Met Office, up to 2019^15^ |

min = minimum; max = maximum; P25 = 25^th^ percentile; P75 = 75^th^ percentile

**Table S3:** Associations (OR 95% CI) between daily pollen levels and asthma hospital admissions, further adjusted for daily NO_2_, PM_2.5_ mass and ozone concentrations

| **Pollen** | **Main model** | **Adjusted for NO_2_** | **Adjusted for PM_2.5_** | **Adjusted for ozone** |
| --- | --- | --- | --- | --- |
| Alder | 1.51 [1.10, 2.09] | 1.54 [1.11, 2.15] | 1.53 [1.10, 2.12] | 1.56 [1.11, 2.18] |
| Birch | 0.93 [0.68, 1.27] | 0.93 [0.67, 1.30] | 0.91 [0.65, 1.27] | 0.95 [0.68, 1.32] |
| Grass | 1.31 [1.00, 1.70] | 1.24 [0.92, 1.65] | 1.25 [0.93, 1.67] | 1.22 [0.91, 1.63] |
| Oak | 1.08 [0.75, 1.56] | 1.03 [0.70, 1.51] | 1.01 [0.69, 1.49] | 1.08 [0.73, 1.58] |
| Nettle | 0.99 [0.78, 1.25] | 0.94 [0.72, 1.23] | 0.94 [0.72, 1.23] | 0.95 [0.73, 1.24] |
| Overall | 1.17 [0.99, 1.37] | 1.14 [0.96, 1.36] | 1.13 [0.95, 1.35] | 1.15 [0.96, 1.37] |

**Table S4:** Associations (OR 95% CI) between daily pollen levels on lag day 1, 2 and 3, as well as cumulative lag from 0-3 days, and asthma hospital admissions

| **Pollen** | **Lag1** | **Lag2** | **Lag3** | **Lag 0-3** |
| --- | --- | --- | --- | --- |
| Alder | 1.33 [0.96, 1.82] | 1.07 [0.75, 1.51] | 0.91 [0.63, 1.33] | 1.09 [0.98, 1.22] |
| Birch | 0.98 [0.72, 1.35] | 1.28 [0.94, 1.75] | 1.08 [0.77, 1.50] | 1.04 [0.94, 1.15] |
| Grass | 1.31 [0.92, 1.89] | 1.01 [0.69, 1.47] | 1.12 [0.77, 1.62] | 1.07 [0.98, 1.18] |
| Oak | 1.10 [0.85, 1.41] | 0.97 [0.75, 1.26] | 0.92 [0.71, 1.20] | 1.04 [0.92, 1.18] |
| Nettle | 1.21 [1.03, 1.42] | 1.07 [0.90, 1.26] | 0.96 [0.80, 1.14] | 1.01 [0.91, 1.12] |
| Overall | 1.33 [0.96, 1.82] | 1.07 [0.75, 1.51] | 0.91 [0.63, 1.33] | 1.06 [1.00, 1.12] |

**Table S5:** Associations (OR 95% CI) between daily pollen levels and asthma hospital admissions, restricted to groups and periods of interest

| **Pollen** | **With reported asthma doctor diagnosis**  **(N events = 1398)** | **With asthma or atopy record in GP data**^†^  **(N events = 724)** | **No acute respiratory infection**^‡^  **(N events = 1862)** | **Repeat hospital admissions excluded**  **(N events = 1313)** | **In ‘high season’**^§^ | **Before Jan 1^st^ 2020 / COVID pandemic**  **(N events = 1522)** |
| --- | --- | --- | --- | --- | --- | --- |
| Alder | 1.51 [1.04, 2.20] | 1.75 [1.01, 3.06] | 1.55 [1.12, 2.14] | 1.56 [1.07, 2.27] | 1.52 [1.10, 2.10] | 1.54 [1.11, 2.15] |
| Birch | 1.00 [0.70, 1.42] | 1.03 [0.62, 1.71] | 0.90 [0.66, 1.24] | 0.77 [0.53, 1.11] | 0.90 [0.65, 1.23] | 0.94 [0.68, 1.3] |
| Grass | 1.21 [0.89, 1.64] | 1.21 [0.79, 1.86] | 1.31 [1.00, 1.71] | 1.34 [0.98, 1.83] | 1.32 [1.01, 1.72] | 1.23 [0.92, 1.65] |
| Oak | 1.21 [0.80, 1.85] | 0.60 [0.31, 1.15] | 1.09 [0.75, 1.57] | 1.39 [0.92, 2.11] | 1.07 [0.74, 1.55] | 1.03 [0.71, 1.51] |
| Nettle | 0.95 [0.72, 1.26] | 1.11 [0.75, 1.65] | 0.98 [0.77, 1.24] | 1.13 [0.85, 1.50] | 1.01 [0.78, 1.29] | 0.94 [0.72, 1.23] |
| Overall | 1.20 [1.00, 1.45] | 1.15 [0.89, 1.49] | 1.17 [1.00, 1.38] | 1.25 [1.04, 1.52] | na | 1.14 [0.96, 1.35] |

^†^Available for ~45% of the UK Biobank cohort only; ^‡^Defined as any of 460-466 ICD9 and J00-06, J20-21 ICD10; ^§^Defined as January to May for alder, March to May for birch, March to August for grass, March to July for oak and May to September for nettles. Number of asthma events was 1195, 672, 1178, 998, 921 for alder, birch, grass, oak and nettle, respectively; na = not applicable

**
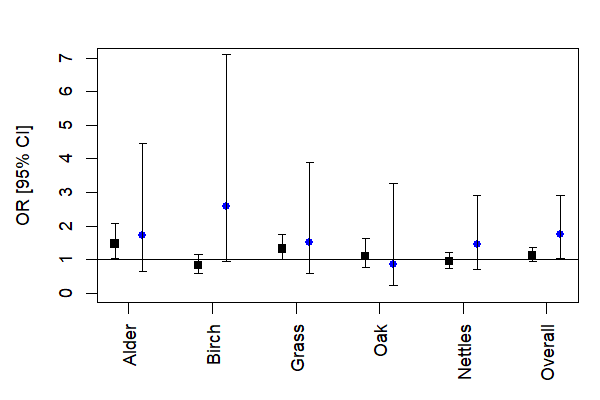
**

**Figure S1:** Associations between the pollen types and asthma hospital admissions, stratified by ethnicity (White = black squares, Non-White = blue circles).

**
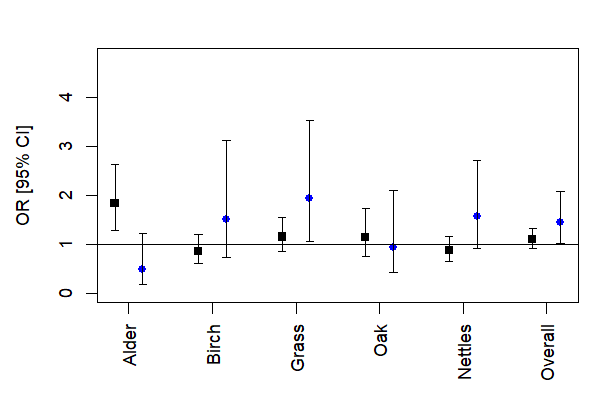
**

**Figure S2:** Associations between the pollen types and asthma hospital admissions, stratified by the 20^th^ vs 80^th^ percentiles of a genetic risk score for atopy (less genetic risk = black squares, greater genetic risk = blue circle)

**REFERENCES**

1. Neal LS, Brown K, Agnew P, et al. Development and verification of a taxa-specific gridded pollen modelling system for the UK. *Aerobiologia*. 2025;1-26. doi: 10.1007/s10453-025-09858-w

2. Navidi W, Weinhandl E. Risk set sampling for case-crossover designs. *Epidemiology*. 2002;13(1):100.

3. Maclure M. The case-crossover design: a method for studying transient effects on the risk of acute events. *Am J Epidemiol*. 2017;185(11):1174-1183. doi:10.1093/aje/kwx105

4. Lu Y, Zeger SL. On the equivalence of case-crossover and time series methods in environmental epidemiology. *Biostatistics*. 2007;8(2):337-344. doi:10.1093/biostatistics/kxl013

5. Jaakkola JJK. Case-crossover design in air pollution epidemiology. *Eur Respir J*. 2003;21(40 suppl):81s-85s. doi:10.1183/09031936.03.00402703

6. Ramasamy A, Curjuric I, Coin LJ, et al. A genome-wide meta-analysis of genetic variants associated with allergic rhinitis and grass sensitization and their interaction with birth order. *J Allergy Clin Immun*. 2011;128(5):996-1005. doi:10.1016/j.jaci.2011.08.030

7. Bønnelykke K, Matheson MC, Pers TH, et al. Meta-analysis of genome-wide association studies identifies ten loci influencing allergic sensitization. *Nat Genet*. 2013;45(8):902-906. doi:10.1038/ng.2694

8. Noguchi E, Morii W, Kitazawa H, et al. A genome-wide meta-analysis reveals shared and population-specific variants for allergic sensitization. *J Allergy Clin Immun*. Published online 2024:S0091674924012855. doi:10.1016/j.jaci.2024.11.033

9. Waage J, Standl M, Curtin JA, et al. Genome-wide association and HLA fine-mapping studies identify risk loci and genetic pathways underlying allergic rhinitis. *Nat Genet*. 2018;50(8):1072-1080. doi:10.1038/s41588-018-0157-1

10. Morii W, Kasai K, Nakamura T, et al. A genome-wide association study for allergen component sensitizations identifies allergen component–specific and allergen protein group–specific associations. *J Allergy Clin Immun: Global*. 2023;2(2):100086. doi:10.1016/j.jacig.2023.100086

11. Sugier PE, Brossard M, Sarnowski C, et al. A novel role for ciliary function in atopy: ADGRV1 and DNAH5 interactions. *J Allergy Clin Immun*. 2018;141(5):1659-1667.e11. doi:10.1016/j.jaci.2017.06.050

12. Zock JP, Heinrich J, Jarvis D, et al. Distribution and determinants of house dust mite allergens in Europe: The European Community Respiratory Health Survey II. *J Allergy Clin Immun*. 2006;118(3):682-690. doi:10.1016/j.jaci.2006.04.060

13. CEDA Archive. HadUK-Grid gridded and regional average climate observations for the UK. Accessed March 15, 2024. https://catalogue.ceda.ac.uk/uuid/4dc8450d889a491ebb20e724debe2dfb

14. Muñoz Sabater J. ERA5-Land hourly data from 1950 to present. Copernicus Climate Change Service (C3S) Climate Data Store (CDS). Accessed: 2024-08-15. Published online 2019. doi:10.24381/cds.e2161bac

15. Savage NH, Agnew P, Davis LS, et al. Air quality modelling using the Met Office Unified Model (AQUM OS24-26): model description and initial evaluation. *Geosci Model Dev*. 2013;6(2):353-372. doi:10.5194/gmd-6-353-2013
